# How Many Criteria Should be Required to Define the DSM-5 Mixed Features Specifier in Depressed Patients?

**DOI:** 10.1192/j.eurpsy.2024.621

**Published:** 2024-08-27

**Authors:** M. Zimmerman

**Affiliations:** Psychiatry, Brown University, Providence, United States

## Abstract

**Introduction:**

During the past 2 decades there has been intense interest in the clinical significance of the concurrence of manic symptoms in depressed patients. DSM-5 introduced a mixed features specifier for both bipolar depression and major depressive disorder. Studies of the DSM-5 mixed features specifier have generally found a low prevalence of mixed depression. One approach towards increasing the sensitivity of the DSM-5 mixed features criteria is to lower the classification threshold.

**Objectives:**

In the present study we examine the impact of lowering the DSM-5 diagnostic threshold from 3 to 2 criteria on the prevalence and validity of the DSM-5 mixed features specifier for depression.

**Methods:**

Four hundred fifty-nine psychiatric patients in a depressive episode were interviewed by a trained diagnostic rater who administered semi-structured interviews including the DSM-5 Mixed Features Specifier Interview. The patients were rated on clinician rating scales of depression, anxiety and irritability, and measures of psychosocial functioning, suicidality, and family history of bipolar disorder.

**Results:**

If the DSM-5 diagnostic threshold is lowered from 3 to 2 symptoms, then the prevalence of mixed features based on the DSM-5 majority of episode time frame tripled from 3.9% to 13.1% (n=60). Based on a past week time frame prevalence more than doubled from 9.4% to 22.9% (n=105) going from the 2 and 3 symptom threshold, respectively. There was no difference between the patients with 2 mixed features and patients with 0 or 1 mixed features on family history of bipolar disorder, psychosocial impairment, presence of comorbid disorders, age of onset, history of suicide attempts or psychiatric hospitalization.

**Conclusions:**

The results of the present study do not support lowering the DSM-5-TR diagnostic threshold for the mixed features specifier in depressed patients from 3 to 2.

**Disclosure of Interest:**

None Declared

